# Genetic Characterization of Peste des Petits Ruminants Virus, Sierra Leone

**DOI:** 10.3201/eid1801.111304

**Published:** 2012-01

**Authors:** Muhammad Munir, Siamak Zohari, Roland Suluku, Neil LeBlanc, Saidu Kanu, Francis A.-R. Sankoh, Mikael Berg, Mohamed L. Barrie, Karl Ståhl

**Affiliations:** Swedish University of Agricultural Sciences, Uppsala, Sweden (M. Munir, S. Zohari, M. Berg, K. Ståhl);; National Veterinary Institute, Uppsala (S. Zohari, N. LeBlanc, K. Ståhl);; Njala University, Freetown, Sierra Leone (R. Suluku, S. Kanu);; Ministry of Agriculture, Forestry and Food Security, Freetown (F.A.-R. Sankoh);; Teko Central Veterinary Laboratory, Makeni, Sierra Leone (M.L. Barrie)

**Keywords:** viruses, Peste des Petits ruminants virus, Peste des Petits ruminants genetic characterization, ruminants, goats, sheep, Sierra Leone

**To the Editor:** Peste des petits ruminants (PPR) is a highly infectious disease of small ruminants, characterized by high rates of illness and death and caused by a single-stranded RNA virus (peste des petits ruminants virus [PPRV]). PPRV can be divided into 4 genetically distinct lineages based on the nucleocapsid (*N*) gene (*1*). The lineages correlate well with geographic distribution of the virus, with lineages I and II mainly restricted to western and central Africa, lineage III to eastern Africa and the Arabian peninsula, and lineage IV to Southeast Asia, the Middle East, and more recently northern Africa (*2*).

PPRV is endemic to most of western Africa, and considered a major constraint on the livestock industry. In Sierra Leone, a country bordered by Guinea, Liberia, and the Atlantic Ocean, and having high goat and sheep populations, PPRV is believed to be the cause of outbreaks of respiratory disease with high death rates. Inadequate veterinary infrastructure and diagnostic capacity, exacerbated by the civil war in 1991–2002, however, has prevented confirmation. In this study, we confirmed presence of PPRV in Sierra Leone, which led to the official report of PPR to the World Organisation for Animal Health (Paris, France).

The study was conducted in April 2009 as part of a training mission organized at Teko Central Veterinary Laboratory, Makeni, Sierre Leone, by the World Organisation for Animal Health Collaborating Centre for Biotechnology–based Diagnosis of Infectious Diseases in Veterinary Medicine (www.sva.se/oie-cc) in collaboration with the Food and Agriculture Organization–Emergency Center for Transboundary Animal Diseases, Bamako, Mali. During the training, blood and serum samples were collected from goats (n = 9) and sheep (n = 1) from 2 smallholders with suspected outbreaks of PPR in the area around Makeni in central Sierra Leone. In addition, serum from 5 goats with respiratory disease was sampled at a livestock market in Kabala 100 km north of Makeni.

Serologic testing was performed at Teko. All serum samples (n = 15) were tested for PPRV antibodies by using a commercial ELISA (BDSL, Ayrshire, UK; *3*); 12 (80%) of the samples were positive for PPRV.

Blood samples were collected on Nobuto filter strips (Advantec MFS Inc., Tokyo, Japan) and transported to the BioSafety Level 3 laboratory at the National Veterinary Institute, Uppsala, Sweden, for nucleic acid detection (*4**,**5*). RNA was eluted from the blood impregnated filter strips and screened for PPRV by using real-time RT-PCR specific for the *N* gene (*6*). Viral RNA was detected in 13 (87%) of the samples, with most of the positive samples indicating high viral load (cycle threshold <20).

For determination of the genetic lineage of detected viruses, RNA from all samples was subjected to PCR amplification of a 351-bp segment of the *N* gene by using the NP3/NP4 primer pair (*7*), but with a modified protocol using the One-Step RT-PCR kit (QIAGEN, Hilden, Germany) (*5*). Amplified PCR products were separated by electrophoresis, gel extracted, purified, and processed for sequencing by using ABI PRISM BigDye Terminator v3.1 kit (Applied Biosystems, Foster City, CA, USA), according to the manufacturer’s instructions.

*N* gene sequences were obtained from 10 (67%) of the samples, and showed 83%–100% nt identity level compared with sequences available in GenBank using the BLASTn tool (www.ncbi.nlm.nih.gov/blast) and 93%–100% identity between each other. Phylogenetic analysis was performed with 4 representative sequences (GenBank accession nos. JN602079–JN602082^)^ from this study by using neighbor-joining and the Kimura 2-parameter model in MEGA5 (CEMI, Tempe, AZ, USA), including *N* gene sequences representing all 4 lineages.

The PPR viruses from Sierra Leone clustered in lineage II with viruses from Mali, Nigeria, and Ghana, and could further be distinguished into 2 clusters (Figure). One virus from Kabala clustered closely with viruses from Mali (Mali 99/1), whereas all others showed 100% identity with a virus from Nigeria (Nig/75/1), in many countries used as vaccine virus strain. In Sierra Leone at the time, however, PPR vaccination was not being performed, suggesting that obtained sequences originated from circulating field viruses related to Nig/75/1 rather than being vaccine derived. This suggestion was strongly supported by the clinical presentation typical of PPR. Surprisingly, no relationship was found with PPRV strains so far described from Guinea, the immediate neighboring country and closest livestock trading partner, or those from Senegal, Guinea-Bissau, Côte d’Ivoire, and Burkina Faso, which constitute lineage I.

**Figure:**
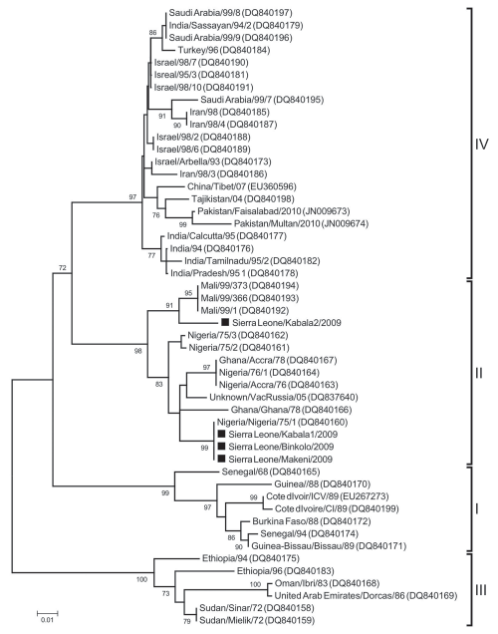
Majority rule consensus tree of peste des petits ruminants viruses (PPRV) based on the variable region of the *N* gene (255 bp), constructed using the neighbor-joining method and the Kimura-2-parameter model in MEGA5 (www.megasoftware.net). Numbers indicate the bootstrap values (2,000 replicates); only values >70% are shown. Horizontal distances are proportional to sequence distances. The figure indicates a clear division of the 4 lineages of PPRV and the sequences obtained in this study clustered in lineage II and are marked with black squares. Scale bar indicates nucleotide substitutions per site.

In conclusion, we confirm the presence of PPRV in Sierra Leone, and provide genetic characterization of detected viruses, knowledge that is fundamental for control, prevention, and in the long run, eradication of the disease. The detection of 2 different sublineages at the livestock market in Kabala shows how markets can serve as mixing vessels, and also gives evidence of at least 2 separate introductions of PPRV into the country, underlining the transboundary nature of the disease, particularly in regions with uncontrolled livestock movements. Since this study, an official vaccination program based on Nigeria/75/1 has been launched.
